# Bayesian estimation of genetic parameters for multivariate threshold and continuous phenotypes and molecular genetic data in simulated horse populations using Gibbs sampling

**DOI:** 10.1186/1471-2156-8-19

**Published:** 2007-05-09

**Authors:** Kathrin F Stock, Ottmar Distl, Ina Hoeschele

**Affiliations:** 1 Institute for Animal Breeding and Genetics, University of Veterinary Medicine Hannover (Foundation), Buenteweg 17p, D-30559 Hannover, Germany; 2 Virginia Bioinformatics Institute and Department of Statistics, Virginia Polytechnic Institute and State University, Blacksburg, VA 24061, USA

## Abstract

**Background:**

Requirements for successful implementation of multivariate animal threshold models including phenotypic and genotypic information are not known yet. Here simulated horse data were used to investigate the properties of multivariate estimators of genetic parameters for categorical, continuous and molecular genetic data in the context of important radiological health traits using mixed linear-threshold animal models via Gibbs sampling. The simulated pedigree comprised 7 generations and 40000 animals per generation. Additive genetic values, residuals and fixed effects for one continuous trait and liabilities of four binary traits were simulated, resembling situations encountered in the Warmblood horse. Quantitative trait locus (QTL) effects and genetic marker information were simulated for one of the liabilities. Different scenarios with respect to recombination rate between genetic markers and QTL and polymorphism information content of genetic markers were studied. For each scenario ten replicates were sampled from the simulated population, and within each replicate six different datasets differing in number and distribution of animals with trait records and availability of genetic marker information were generated. (Co)Variance components were estimated using a Bayesian mixed linear-threshold animal model via Gibbs sampling. Residual variances were fixed to zero and a proper prior was used for the genetic covariance matrix.

**Results:**

Effective sample sizes (ESS) and biases of genetic parameters differed significantly between datasets. Bias of heritability estimates was -6% to +6% for the continuous trait, -6% to +10% for the binary traits of moderate heritability, and -21% to +25% for the binary traits of low heritability. Additive genetic correlations were mostly underestimated between the continuous trait and binary traits of low heritability, under- or overestimated between the continuous trait and binary traits of moderate heritability, and overestimated between two binary traits. Use of trait information on two subsequent generations of animals increased ESS and reduced bias of parameter estimates more than mere increase of the number of informative animals from one generation. Consideration of genotype information as a fixed effect in the model resulted in overestimation of polygenic heritability of the QTL trait, but increased accuracy of estimated additive genetic correlations of the QTL trait.

**Conclusion:**

Combined use of phenotype and genotype information on parents and offspring will help to identify agonistic and antagonistic genetic correlations between traits of interests, facilitating design of effective multiple trait selection schemes.

## Background

Use of linear models for the estimation of genetic parameters for categorical traits violates basic assumptions of mixed linear model methodology. Algorithms have been developed to transform linear model estimates to the underlying liability scale in order to compensate for the estimation bias caused by analysis of non-linear traits in linear models, but transformed genetic parameter estimates might still be significantly biased [e.g., [1-4]]. Use of threshold models for estimation of genetic parameters directly accounts for the non-linear nature of categorical traits, and threshold model estimates should be more reliable than linear model estimates or transformed linear model estimates [e.g., [5-7]].

Markov chain Monte Carlo (MCMC) methods such as Gibbs sampling (GS) make it feasible to implement multivariate threshold models or multivariate mixed linear-threshold models. Animal models fully use the available pedigree information, but accuracy of genetic variance and covariance estimates and convergence of GS chains might be a problem in the case of low trait prevalences and few observations per individual [e.g., [5,8-10]]. Therefore, in practical situations implementation of multivariate animal threshold models is not always straightforward. The inclusion of continuous traits, i.e. use of a multivariate linear-threshold model, is expected to increase the reliability of genetic parameter estimates [e.g., [2,11-13]].

In animal breeding, health data are often recorded using discrete categories, whilst most performance traits are continuous. In the horse, binary coding has been used for investigations on radiographic findings in the equine limbs, and high prevalences of radiologically visible alterations, mostly in the range of 10–25%, have been determined in the limbs of young horses [e.g. [14-17]]. This promoted the search for preventive rather than curative measures. Because strength and soundness of the equine locomotory system is of great importance in all sectors of the horse industry, inclusion of radiological health traits in current breeding schemes of the Warmblood riding horse has been suggested [18,19]. Reliably estimated genetic parameters for these categorical traits provide the basis to do so. For the analyzed radiographic health data, applicability of transformation factors according to Dempster and Lerner [20] and Vinson et al. [21] to linear animal model estimates obtained with residual maximum likelihood (REML) has been proven via simulation [22]. However, rate of over- or underestimation, which is caused by analysis of binary traits in linear models and has to be compensated via transformation, depends on data structure. Re-evaluation of transformation procedure will therefore be required for analyses of data of different structure with respect to distribution and kind of available information. Implementation of a Bayesian multivariate animal threshold or mixed linear-threshold model for (co)variance component estimation may provide a worthwhile alternative.

Quantitative trait loci (QTL), i.e. genome regions which include genes that influence the phenotype of an individual with respect to a particular trait, have been identified for production and health traits in different species [23]. Increasing knowledge on genetic determination of radiological health traits [24,25] implies further opportunities for improvement of genetic evaluation and selection schemes in the horse. Conditions for efficient use of marker-assisted selection have been described [26], but the effects of combined use of phenotypic and genotypic data on the accuracy of genetic parameter estimation for categorical traits has not been studied yet. Requirements for successful implementation of multivariate animal threshold models including phenotypic and genotypic information are unknown.

The aim of this study was to characterize the properties of multivariate estimation of genetic parameters for categorical, continuous and molecular genetic data using linear-threshold animal models and Gibbs sampling. Impact of data structure and quality of molecular genetic data on the accuracy of genetic parameter estimates was studied in the context of important radiological health traits in Warmblood horses.

## Results

### Gibbs chains

The number of rounds of Gibbs sampling that had to be discarded as burn-in ranged between 5000 and 53000. Mean lengths of burn-in ranged between 8100 and 13866 rounds in the analyses of the different datasets. Mean, minimum and maximum length of burn-in were lowest in the analyses of dataset A2 and highest in the analyses of dataset B1.

Mean, minimum and maximum ESS of heritabilities and additive genetic correlations by dataset and quality of genotype information on T2 are given in Tables 1 and 2. For heritability of the continuous trait (T1) ESS ranged between 286.9 and 858.4 in the analyses of data on animals from one generation (datasets A1, B1 and C1) and between 1222.7 and 1949.0 in the analyses of data on animals from two subsequent generations (datasets A2, B2 and C2). For heritability of the binary traits ESS ranged between 23.0 and 139.3 for T2, between 24.1 and 147.5 for T3, between 15.4 and 115.2 for T4, and between 50.6 and 289.6 for T5 in the analyses of all datasets. Mean ESS was 810.7 to 1325.4 for the additive genetic correlation between T1 and T5, 175.7 to 500.7 for the additive genetic correlations of T1 with T2, T3 and T4, and 86.4 to 376.2 for the additive genetic correlation between traits T2, T3, T4 and T5. Significant influence on ESS was determined for the analyzed dataset (P < 0.001), but not for the quality of genetic marker information. There were no significant differences between ESS for all heritabilities and all additive genetic correlations in analyses of datasets B1 and C1 and between ESS for all heritabilities and all additive genetic correlations but r_g12 _in analyses of datasets B2 and C2.

**Table 1:** Mean (minimum, maximum) effective sample size of estimated heritabilities for investigated QTL scenarios.

	Dataset					
	
Parameter	A1	A2	B1	B2	C1	C2
h^2^_1_	641.7 (533.5, 778.6)	1567.9 (1341.7, 1791.7)	455.6 (297.7, 608.1)	1631.9 (1426.4, 1782.9)	505.3 (402.4, 581.6)	1620.5 (1417.3, 1753.0)
	652.5 (504.5, 829.9)	1562.8 (1266.4, 1749.8)	480.7 (366.5, 611.9)	1659.3 (1407.7, 1932.8)	472.7 (301.1, 643.0)	1640.8 (1330.0, 1881.2)
	628.1 (371.6, 858.4)	1590.1 (1236.2, 1713.0)	451.5 (286.9, 671.3)	1630.9 (1222.7, 1888.9)	453.5 (347.8, 626.7)	1655.6 (1487.9, 1949.0)
h^2^_2_	93.8 (36.4, 117.9)	94.6 (76.5, 122.9)	75.4 (46.5, 113.4)	93.8 (48.3, 139.3)	66.6 (36.6, 105.8)	102.2 (62.8, 129.2)
	86.5 (59.6, 119.8)	95.9 (56.5, 119.5)	75.4 (48.3, 124.6)	95.9 (56.5, 119.5)	72.9 (23.0, 90.7)	105.2 (83.6, 120.9)
	69.8 (42.7, 100.7)	99.8 (31.3, 133.5)	84.7 (55.4, 114.6)	97.4 (53.1, 124.8)	76.4 (46.7, 112.3)	100.1 (75.5, 120.5)
h^2^_3_	79.0 (25.9, 113.0)	103.8 (62.8, 147.5)	60.6 (35.1, 89.4)	96.1 (71.0, 123.5)	58.5 (29.1, 77.3)	84.8 (58.9, 102.4)
	68.7 (30.7, 105.5)	92.6 (76.5, 113.9)	65.7 (41.6, 92.3)	92.6 (76.5, 113.9)	58.7 (37.9, 78.0)	81.2 (42.3, 117.4)
	83.9 (49.6, 113.7)	101.4 (63.7, 142.0)	44.0 (24.1, 59.6)	91.8 (61.8, 128.5)	53.4 (35.5, 75.2)	91.6 (54.1, 141.7)
h^2^_4_	53.4 (31.5, 77.2)	75.5 (55.3, 95.1)	61.8 (30.7, 79.3)	75.3 (48.6, 98.7)	58.9 (34.8, 89.9)	82.0 (46.2, 115.2)
	57.2 (34.3, 108.0)	77.1 (40.3, 99.8)	58.5 (15.4, 84.8)	77.1 (40.3, 99.8)	63.3 (35.1, 110.5)	82.8 (64.6, 114.6)
	76.2 (50.8, 102.5)	68.4 (24.6, 105.4)	68.0 (50.0, 98.9)	75.2 (50.2, 102.3)	57.1 (47.8, 81.1)	70.9 (37.0, 91.5)
h^2^_5_	131.1 (71.7, 179.7)	195.5 (75.9, 251.2)	108.9 (50.6, 143.0)	216.7 (145.1, 259.7)	117.7 (85.3, 162.4)	207.1 (159.8, 284.2)
	121.8 (79.5, 167.8)	204.7 (133.4, 277.6)	106.7 (66.0,131.5)	204.7 (133.4, 277.6)	113.5 (88.4, 148.0)	198.4 (100.8, 289.6)
	129.3 (95.9, 174.1)	206.7 (147.0, 276.6)	113.6 (50.8, 146.5)	209.9 (74.2, 272.0)	101.4 (62.6, 142.9)	227.1 (141.5, 274.9)

QTL: quantitative trait locus;

h^2^_1_: heritability of the continuous trait T1; h^2^_2_(h^2^_3_, h^2^_4_, h^2^_5_): heritability of binary trait T2 (T3, T4, T5);

QTL scenarios: different scenarios with respect to recombination rate between markers and QTL (r) and polymorphism information content (PIC) of markers with r = 0.00 and PIC = 0.9 in the first line, r = 0.01 and PIC = 0.9 in the second line, and r = 0.00 and PIC = 0.7 in the third line.

For details on datasets A1 to C2 see Table 6.

**Table 2:** Mean (minimum, maximum) effective sample size of estimated additive genetic correlations for investigated QTL scenarios.

	Dataset					
	
Parameter	A1	A2	B1	B2	C1	C2
r_g12_	435.7 (306.8, 639.8)	475.2 (198.2, 794.3)	367.1 (158.7, 612.5)	444.1 (238.1, 576.8)	281.3 (210.9, 416.0)	314.6 (240.9, 451.0)
	464.0 (191.0, 701.5)	485.1 (313.2, 710.0)	287.8 (148.9, 511.4)	388.9 (209.6, 566.3)	284.6 (202.4, 400.7)	337.0 (172.1, 445.7)
	347.2 (222.0, 482.4)	448.2 (326.1, 669.8)	316.2 (196.6, 471.0)	373.2 (242.0, 494.0)	309.1 (124.4, 591.1)	346.4 (257.6, 474.2)
r_g13_	383.8 (221.8, 497.4)	500.7 (217.6, 658.0)	236.4 (139.3, 339.3)	436.7 (217.5, 603.2)	207.9 (100.9, 371.0)	430.9 (201.5, 650.8)
	384.6 (75.2, 662.9)	498.5 (242.5, 758.7)	278.4 (107.3, 538.6)	453.6 (219.5, 664.0)	222.9 (102.9, 346.8)	459.5 (238.8, 652.6)
	353.4 (181.9, 523.0)	512.0 (286.6, 814.1)	230.3 (121.0, 435.1)	463.5 (244.3, 693.0)	213.9 (111.5, 442.5)	449.4 (194.5, 723.8)
r_g14_	257.1 (127.9, 371.0)	293.3 (194.5, 493.1)	175.7 (52.1, 261.0)	282.8 (147.2, 459.2)	198.5 (90.8, 295.2)	281.5 (158.0, 389.1)
	291.2 (191.9, 514.9)	336.8 (161.9, 533.1)	213.5 (151.9, 290.1)	274.9 (174.5, 369.1)	182.8 (128.6, 228.0)	284.2 (111.2, 419.9)
	278.3 (170.7, 470.7)	312.3 (139.6, 526.8)	211.2 (126.2, 260.9)	261.7 (123.5, 405.3)	207.9 (108.3, 301.0)	245.5 (172.3, 334.4)
r_g15_	1056.4 (722.0, 1399.0)	1301.3 (1063.7, 1489.8)	836.1 (479.0, 1471.4)	1312.6 (1065.5, 1492.6)	841.7 (482.2, 1299.6)	1237.8 (815.4, 1399.2)
	1043.5 (638.6, 1453.7)	1312.0 (1141.2, 1614.4)	810.7 (553.7, 1308.4)	1315.6 (1005.0, 1418.0)	900.0 (658.3, 1460.2)	1325.4 (1061.5, 1547.2)
	1049.5 (614.2, 1469.9)	1313.5 (1069.9, 1614.0)	905.2 (739.5, 1245.4)	1287.0 (1108.5, 1368.9)	852.5 (593.2, 1207.6)	1281.6 (1027.8, 1545.3)
r_g23_	149.4 (129.6, 187.4)	159.3 (76.5, 197.8)	121.6 (82.2, 162.2)	155.5 (112.9, 208.3)	104.5 (69.2, 125.8)	130.7 (75.4, 179.4)
	168.0 (153.1, 201.9)	157.8 (108.9, 196.8)	117.3 (54.7, 161.9)	157.2 (80.2, 212.2)	128.6 (89.1, 156.0)	152.1 (94.6, 220.7)
	139.2 (87.9, 204.7)	145.9 (90.5, 199.8)	121.6 (71.1, 188.7)	124.7 (81.1, 161.8)	118.8 (81.9, 176.5)	148.6 (106.2, 187.6)
r_g24_	107.1 (74.4, 142.7)	104.5 (42.2, 155.4)	97.9 (41.4, 139.5)	104.7 (74.9, 143.5)	91.4 (45.3, 112.1)	103.9 (85.9, 129.5)
	97.9 (32.1, 161.2)	124.8 (94.2, 192.8)	87.1 (30.8, 117.9)	126.8 (71.8, 169.2)	87.5 (60.2, 153.1)	112.4 (75.3, 155.1)
	94.6 (53.9, 146.4)	107.4 (60.5, 132.0)	100.0 (63.1, 121.1)	104.2 (45.4, 140.9)	92.7 (65.0, 126.8)	112.9 (91.3, 158.3)
r_g25_	215.7 (180.3, 292.2)	256.0 (214.7, 331.0)	170.8 (128.5, 230.0)	258.8 (176.2, 342.7)	143.9 (65.1, 185.5)	196.9 (158.0, 223.6)
	206.7 (93.5, 297.3)	259.1 (151.4, 329.2)	190.4 (84.7, 288.8)	230.4 (179.1, 335.9)	155.1 (84.1, 257.9)	219.3 (128.8, 273.7)
	196.0 (105.3, 290.8)	216.2 (157.4, 299.3)	166.0 (131.6, 215.1)	225.9 (161.6, 281.0)	161.5 (92.6, 251.9)	241.7 (196.6, 310.3)
r_g34_	112.9 (83.9, 189.5)	134.9 (81.7, 180.1)	86.4 (33.3, 140.4)	105.0 (52.5, 162.0)	91.2 (57.2, 132.3)	119.2 (89.8, 148.6)
	123.8 (78.8, 149.8)	133.1 (81.0, 184.9)	90.6 (58.4, 127.4)	117.8 (89.1, 157.2)	92.8 (80.0, 112.5)	115.6 (71.1, 143.3)
	120.7 (63.5, 163.1)	121.4 (60.5, 159.8)	94.4 (53.4, 131.3)	132.5 (80.7, 168.4)	92.3 (45.1, 144.0)	128.8 (52.8, 179.2)
r_g35_	250.0 (174.0, 351.5)	376.2 (281.8, 491.5)	218.2 (103.7, 317.9)	301.7 (154.3, 445.0)	190.8 (106.4, 285.9)	338.5 (241.1, 418.1)
	286.5 (220.8, 336.0)	356.4 (224.6, 473.6)	208.2 (112.5, 332.5)	316.1 (194.6, 443.7)	179.7 (80.9, 310.7)	342.1 (264.2, 420.0)
	273.9 (184.2, 387.0)	351.8 (271.0, 443.1)	215.6 (149.6, 336.8)	325.8 (152.3, 398.5)	196.6 (98.8, 269.4)	332.8 (266.5, 399.4)
r_g45_	164.7 (83.0, 236.4)	190.0 (109.6, 272.5)	120.1 (52.0, 180.9)	184.8 (117.0, 247.4)	122.2 (82.3, 167.6)	145.9 (112.3, 182.1)
	159.8 (91.2, 221.1)	181.0 (83.9, 239.5)	115.2 (73.3, 167.4)	155.8 (90.9, 259.2)	135.6 (87.0, 192.4)	164.5 (117.2, 223.2)
	163.6 (123.8, 241.2)	169.6 (131.3, 264.4)	122.5 (92.8, 187.8)	174.2 (67.9, 248.7)	121.0 (70.1, 176.9)	156.8 (104.1, 205.1)

QTL: quantitative trait locus;

r_g12 _(r_g13_, r_g14_, r_g15_): additive genetic correlation between continuous trait T1 and binary trait T2 (T3, T4, T5);

r_g23 _(r_g24_, r_g25_, r_g34_, r_g35_, r_g45_): additive genetic correlations between binary traits T2 and T3 (T2 and T4, T2 and T5, T3 and T4, T3 and T5, T4 and T5);

QTL scenarios: different scenarios with respect to recombination rate between markers and QTL (r) and polymorphism information content (PIC) of markers with r = 0.00 and PIC = 0.9 in the first line, r = 0.01 and PIC = 0.9 in the second line, and r = 0.00 and PIC = 0.7 in the third line.

For details on datasets A1 to C2 see Table 6.

Mean MCE was 0.016 to 0.017 for heritability of T1 in analyses of datasets B1 and C1, and 0.002 to 0.010 for heritability of T1 in analyses of datasets A1, A2, B2 and C2 and heritabilities of the binary traits (T2 to T5) in analyses of all datasets. Mean MCE for additive genetic correlations ranged between 0.001 and 0.004 in all analyses.

### Parameter estimates

Genetic parameter estimates differed little between scenarios r0p9, r1p9 and r0p7. In the analyses of the six datasets ranges of heritability estimates were h^2 ^= 0.487–0.667 for T1, h^2 ^= 0.058–0.171 for T2, h^2 ^= 0.193–0.343 for T3, h^2 ^= 0.081–0.170 for T4, and h^2 ^= 0.210–0.354 for T5. Ranges of estimates for the simulated additive genetic correlations were r_g12 _= 0.009–0.327, r_g13 _= 0.102–0.316, r_g14 _= -0.295 to -0.034, and r_g45 _= -0.506 to 0.150.

Mean, minimum and maximum bias of heritabilities and additive genetic correlations by dataset and quality of genotype information on T2 are given in Tables 3 and 4. Bias of heritability estimates for T1 ranged between -0.075 and 0.221 in the analyses of datasets A1, B1 and C1, and between -0.089 and -0.021 in the analyses of datasets A2, B2 and C2. Mean bias of heritability estimates for T2 was -0.134 to 0.205 in the analyses of datasets A1, A2, B1 and B2, 0.94 to 0.99 in analyses of dataset C1, and 0.47 to 0.52 in analyses of dataset C2. Mean bias of heritability estimates was -0.057 to 0.024 for T3, 0.065 to 0.231 for T4, and -0.018 to 0.095 for T5 in all analyses. Extremes of negative and positive bias of heritability estimates were -0.414 and 1.811 for T2, -0.233 and 0.316 for T3, -0.201 and 0.649 for T4, and -0.156 and 0.423 for T5. Additive genetic correlations between T1 and T2, T1 and T3, and T1 and T4 had a mean bias of -0.503 to 0.177 and a bias range from -0.958 to 0.637. Additive genetic correlation between T4 and T5 had a mean bias of 0.127 to 0.376 and a bias range from -1.748 to 1.532. The analyzed dataset had a significant influence on the bias of heritability estimates for T1, T2, T4 and T5 (P < 0.01) and of estimated additive genetic correlations between T1 and T2, T1 and T3, and T1 and T4 (P < 0.001). Bias of heritability estimate for T2 was further significantly dependent on the quality of genetic marker information (P < 0.01), with lower means in scenario r0p7 than in scenarios r0p9 and r1p9.

**Table 3:** Mean (minimum, maximum) relative bias of estimated heritabilities for investigated QTL scenarios.

	Dataset					
	
Parameter	A1	A2	B1	B2	C1	C2
h^2^_1_	0.061 (-0.013, 0.169)	-0.048 (-0.066, -0.021)	0.024 (-0.075, 0.221)	-0.064 (-0.089, -0.032)	0.025 (-0.065, 0.210)	-0.064 (-0.089, -0.034)
	0.060 (-0.014, 0.165)	-0.048 (-0.065, -0.021)	0.025 (-0.071, 0.218)	-0.064 (-0.087, -0.033)	0.023 (-0.075, 0.213)	-0.064 (-0.087, -0.034)
	0.059 (-0.010, 0.168)	-0.049 (-0.065, -0.020)	0.026 (-0.063, 0.221)	-0.064 (-0.089, -0.032)	0.024 (-0.071, 0.215)	-0.064 (-0.089, -0.033)
h^2^_2_	0.068 (-0.321, 0.460)	-0.049 (-0.390, 0.101)	0.205 (-0.112, 0.733)	-0.020 (-0.289, 0.142)	0.955 (0.599, 1.811)	0.506 (0.233, 0.706)
	0.078 (-0.330, 0.490)	-0.031 (-0.365, 0.134)	0.183 (-0.203, 0.721)	-0.027 (-0.280, 0.118)	0.989 (0.511, 1.724)	0.519 (0.208, 0.696)
	-0.134 (-0.414, 0.171)	-0.213 (-0.415, -0.048)	0.070 (-0.288, 0.551)	-0.161 (-0.336, -0.057)	0.938 (0.361, 1.802)	0.467 (0.219, 0.672)
h^2^_3_	0.013 (-0.218, 0.176)	-0.043 (-0.188, 0.106)	-0.005 (-0.179, 0.304)	-0.051 (-0.198, 0.072)	-0.034 (-0.224, 0.254)	-0.057 (-0.218, 0.067)
	0.019 (-0.205, 0.167)	-0.039 (-0.180, 0.127)	-0.010 (-0.193, 0.326)	-0.052 (-0.207, 0.070)	-0.008 (-0.233, 0.348)	-0.055 (-0.177, 0.049)
	0.024 (-0.185, 0.180)	-0.040 (-0.152, 0.112)	-0.006 (-0.193, 0.361)	-0.041 (-0.179, 0.101)	-0.013 (-0.224, 0.281)	-0.045 (-0.178, 0.082)
h^2^_4_	0.160 (-0.143, 0.434)	0.073 (-0.164, 0.272)	0.195 (-0.076, 0.566)	0.073 (-0.129, 0.243)	0.231 (-0.094, 0.558)	0.087 (-0.135, 0.238)
	0.141 (-0.162, 0.390)	0.065 (-0.090, 0.273)	0.245 (-0.117, 0.649)	0.074 (-0.128, 0.212)	0.226 (-0.072, 0.588)	0.080 (-0.140, 0.240)
	0.150 (-0.201, 0.418)	0.061 (-0.111, 0.273)	0.235 (-0.099, 0.657)	0.090 (-0.151, 0.271)	0.241 (-0.124, 0.630)	0.078 (-0.131, 0.242)
h^2^_5_	-0.018 (-0.143, 0.088)	0.008 (-0.108, 0.102)	0.084 (-0.105, 0.358)	0.066 (-0.064, 0.156)	0.084 (-0.118, 0.392)	0.068 (-0.065, 0.147)
	-0.010 (-0.156, 0.087)	0.007 (-0.099, 0.118)	0.074 (-0.136, 0.363)	0.066 (-0.068, 0.143)	0.084 (-0.125, 0.375)	0.068 (-0.061, 0.157)
	-0.015 (-0.143, 0.081)	0.008 (-0.103, 0.113)	0.090 (-0.108, 0.393)	0.067 (-0.059, 0.143)	0.095 (-0.097, 0.423)	0.066 (-0.071, 0.156)

QTL: quantitative trait locus;

h^2^_1_: heritability of the continuous trait T1; h^2^_2 _(h^2^_3_, h^2^_4_, h^2^_5_): heritability of binary trait T2 (T3, T4, T5);

QTL scenarios: different scenarios with respect to recombination rate between markers and QTL (r) and polymorphism information content (PIC) of markers with r = 0.00 and PIC = 0.9 in the first line, r = 0.01 and PIC = 0.9 in the second line, and r = 0.00 and PIC = 0.7 in the third line.

For details on datasets A1 to C2 see Table 6.

**Table 4:** Mean (minimum, maximum) relative bias of selected additive genetic correlations for investigated QTL scenarios.

	Dataset					
	
Parameter	A1	A2	B1	B2	C1	C2
r_g12_	-0.503 (-0.958, 0.040)	-0.332 (-0.647, 0.204)	-0.312 (-0.859, 0.374)	-0.176 (-0.501, 0.355)	-0.207 (-0.740, 0.416)	-0.082 (-0.417, 0.637)
	-0.488 (-0.925, 0.014)	-0.335 (-0.628, 0.215)	-0.292 (-0.910, 0.304)	-0.179 (-0.486, 0.356)	-0.199 (-0.795, 0.336)	-0.066 (-0.381, 0.624)
	-0.331 (-0.908, 0.131)	-0.198 (-0.575, 0.280)	-0.261 (-0.954, 0.194)	-0.120 (-0.479, 0.448)	-0.247 (-0.936, 0.225)	-0.101 (-0.447, 0.501)
r_g13_	-0.056 (-0.335, 0.360)	-0.081 (-0.481, 0.344)	0.152 (-0.386, 0.543)	-0.017 (-0.430, 0.412)	0.177 (-0.386, 0.581)	-0.017 (-0.449, 0.438)
	-0.060 (-0.320, 0.366)	-0.084 (-0.481, 0.328)	0.159 (-0.368, 0.569)	-0.021 (-0.426, 0.432)	0.164 (-0.392, 0.548)	-0.019 (-0.436, 0.404)
	-0.065 (-0.330, 0.315)	-0.081 (-0.488, 0.328)	0.158 (-0.398, 0.506)	-0.028 (-0.442, 0.398)	0.159 (-0.382, 0.569)	-0.021 (-0.440, 0.376)
r_g14_	-0.494 (-0.904, 0.026)	-0.193 (-0.513, 0.176)	-0.271 (-0.785, 0.194)	0.014 (-0.401, 0.462)	-0.271 (-0.714, 0.169)	0.008 (-0.416, 0.410)
	-0.484 (-0.916, -0.007)	-0.189 (-0.538, 0.153)	-0.297 (-0.804, 0.122)	0.008 (-0.416, 0.464)	-0.268 (-0.774, 0.210)	0.010 (-0.378, 0.470)
	-0.473 (-0.825, 0.031)	-0.187 (-0.533, 0.184)	-0.284 (-0.814, 0.237)	0.006 (-0.413, 0.430)	-0.289 (-0.831, 0.137)	-0.004 (-0.405, 0.456)
r_g45_	0.127 (-0.598, 0.753)	0.291 (-0.426, 0.657)	0.217 (-1.569. 1.366)	0.368 (-0.766, 0.881)	0.211 (-1.660, 1.532)	0.364 (-0.813, 0.923)
	0.140 (-0.567, 0.760)	0.284 (-0.428, 0.729)	0.196 (-1.683, 1.484)	0.374 (-0.818, 0.978)	0.174 (-1.748, 1.499)	0.369 (-0.834, 1.033)
	0.163 (-0.652, 0.795)	0.286 (-0.412, 0.721)	0.232 (-1.616, 1.524)	0.365 (-0.858, 0.928)	0.154 (-1.624, 1.407)	0.376 (-0.806, 0.960)

QTL: quantitative trait locus;

r_g12 _(r_g13_, r_g14_): additive genetic correlation between continuous trait T1 and binary trait T2 (T3, T4);

r_g45_: additive genetic correlations between binary traits T4 and T5;

QTL scenarios: different scenarios with respect to recombination rate between markers and QTL (r) and polymorphism information content (PIC) of markers with r = 0.00 and PIC = 0.9 in the first line, r = 0.01 and PIC = 0.9 in the second line, and r = 0.00 and PIC = 0.7 in the third line.

For details on datasets A1 to C2 see Table 6.

## Discussion

### Mixed linear-threshold model analyses via Gibbs sampling

Simulated categorical, continuous and molecular genetic data were used for multivariate estimation of genetic parameters in mixed linear-threshold animal models via Gibbs sampling. General statements on the properties of multivariate analyses in mixed linear-threshold models require validation using independent sample datasets. For this study, simulation of multiple populations per study scenario and random sampling of one set of datasets per population was straightforward, but computationally expensive. In a preliminary study we therefore tested an alternative approach, with simulation of one population per study scenario and repeated random sampling of sets of datasets from one population. Because results regarding effective sample sizes and biases were not statistically different under these two approaches (results not shown), the computationally less expensive approach was chosen for the main study, i.e. replicate datasets were generated by repeated random sampling from single multi-generation populations.

Datasets used for the analyses differed in the number of animals with trait information, the distribution of animals with trait information and the availability of marker genotype information. The simulated pedigree structure resembled that of the Hanoverian Warmblood horse, and simulated data resembled the distribution of radiographic findings in the limbs of young Warmblood riding horses [18]. The traits of interest were binary, but one continuous trait was included in the multivariate analyses in order to improve mixing and convergence of the Gibbs sampler. Even in the analyses of the smallest datasets burn-in periods of 5000 to 15000 rounds were mostly sufficient for the Gibbs chains to converge. However, effective sample size of heritability of the continuous trait was considerably larger than effective sample sizes of heritabilities and additive genetic correlations of the binary traits. Effective sample sizes mentioned in literature for heritabilities of binary traits and uni- or bivariate animal threshold models were often in the same order despite larger numbers of informative animals and longer chain lengths [27-30]. In a previous simulation study inclusion of a continuous trait in animal threshold model analyses of binary traits had a positive effect on convergence, but not on effective sample size [8]. We found significant dependence of effective sample sizes on amount and distribution of available information. Given a certain number of animals with trait records, effective sample sizes for heritabilities were larger when animals were from two subsequent generations instead of one generation. There was no such clear effect on effective sample sizes for additive genetic correlations. Despite fixation of residual covariances to zero, effective sample sizes for additive genetic covariances and correlations were in many cases larger than effective sample sizes for additive genetic variances and heritabilities of the binary traits. Results from literature are not consistent in this respect [31].

### Genetic parameter estimates

Bias of genetic parameter estimates is the major argument against transformation of linear model estimates and in favor of threshold model analysis of binary traits [e.g. [1,2]]. Heritabilities of the continuous trait and the moderately heritable binary traits (h^2 ^= 0.25) were over- or underestimated by maximally 10 percent. If heritability of the binary trait was lower (h^2 ^= 0.10) and only phenotype information was considered, mean bias increased to up to 25 percent over- or underestimation. These values are in agreement with literature [1,10,32] and clearly smaller than [2] or similar to [22] reported bias of transformed linear model estimates. Inclusion of fixed genotype effect in the model resulted in marked overestimation of heritability of the QTL trait. If trait information was available on animals in only one generation, upward bias was larger than 90 percent, indicating failure to reliably estimate heritability of the polygenic component. Heritability estimates were close to simulated overall heritabilities. If trait information was available on animals in two subsequent generations, i.e. to parents and offspring, upward bias of polygenic heritability estimates was reduced to about 50 percent.

Bias of additive genetic correlations between continuous and binary traits was dependent on heritability of the binary trait, but largely independent of the sign of the simulated correlation. Low heritability of the binary trait (h^2 ^= 0.10) resulted in downward bias of continuous-binary correlation estimates by up to 50 percent, i.e. estimates for both positive and negative additive genetic correlations were closer to zero than the simulated values. Estimates for the positive additive genetic correlation between the continuous and the moderately heritable binary trait (h^2 ^= 0.25) were on average less biased, with upward bias of up to 18 percent in the small datasets and downward bias of up to 8 percent in the larger datasets. Ranges of biases were similar for all continuous-binary correlation estimates. Estimates for the positive additive genetic correlation between two of the binary traits showed considerable variation of bias and were on average overestimated by 13 to 38 percent. Variation of bias was larger if information on animals from only one generation was considered, and overestimation was larger if information on animals from two subsequent generations was considered. Underestimation of positive additive genetic correlation between one continuous trait of moderate heritability (h^2 ^= 0.25) and binary traits of low heritability (h^2 ^= 0.05 or 0.10) and low prevalence (0.05 or 0.15) has been reported previously [33]. However, in this and a similar study estimates for positive additive genetic correlation between the two binary traits were biased downward as well [2,33]. Differences in simulation parameters and method of analysis may be responsible for the different results. In contrast with our study, simulated additive genetic correlations were all 0.5, residual correlations of 0.3 or 0.2 or -0.2 were simulated, and analyses were performed in linear animal model with residual maximum likelihood (REML). Theoretically, estimates for additive genetic correlations should not be affected by violation of assumption of normality when using linear models for analysis of binary data [21,34]. However, opposite directions of biases seem to arise from use of linear and threshold models for estimation of genetic correlations between binary traits.

### Amount and structure of data

Amount and structure of available information had little influence on accuracy of heritability estimates for the continuous trait and the binary traits of moderate heritability. Increase of the amount of available information increased accuracy of heritability estimates for the binary traits of low heritability and of additive genetic correlation estimates between continuous and binary traits, but also increased the upward bias of additive genetic correlation estimates between two binary traits. Influence of quality of genetic marker information on accuracy of heritability estimates for the binary QTL trait and additive genetic correlation estimates between the continuous trait and the binary QTL trait was less distinct. Given high PIC of genetic markers, estimates were largely unaffected by presence or absence of recombination between markers and QTL. Decrease of PIC resulted in lower heritability estimates, i.e. increased negative bias or decreased positive bias of heritability estimates, and larger range of bias of heritability estimates for the QTL trait. Estimates of additive genetic correlations between the continuous trait and the binary QTL trait were least biased when phenotype and genotype information was considered, genotype information included highly informative markers, and trait information referred to animals from two subsequent generations. There are no reports of similar investigations on influences on accuracy of genetic parameter estimates. Simulated heritabilities and additive genetic correlations were aligned with results of previous real data analyses and kept constant in this study. Furthermore, we simulated only four of the ten additive genetic correlations and no residual correlations in the multivariate setting of one continuous and four binary traits. Correlations between the binary QTL trait and another binary trait were not simulated. Impact of quality of genetic marker information on accuracy of additive genetic correlation estimates between two binary traits, one of which is influenced by QTL, requires further investigation. Simulation can be extended by modification of heritability levels, closeness and sign of genetic correlations and inclusion of more than one QTL trait. This study documents general feasibility of combined use of phenotype and genotype data for estimation of genetic parameters in the horse using multivariate mixed linear-threshold animal models and Gibbs sampling.

## Conclusion

It is feasible to perform multivariate estimation of genetic parameters for categorical, continuous and molecular genetic data in mixed linear-threshold animal models via Gibbs sampling using data and pedigree structures similar to those encountered in the Hanoverian Warmblood horse. Use of trait information on two subsequent generations of animals can increase accuracy of parameter estimates more than merely increasing the number of informative animals from one generation. Impact of different quality of genetic marker information with respect to recombination rate and PIC was minor in the scenarios studied. Consideration of marker genotype information as a fixed effect in the model is likely to result in overestimation of polygenic heritability of a QTL trait, but may be advantageous for quantification of additive genetic correlations between traits of interest. Improved identification of agonistic and antagonistic genetic correlations between traits of interests will facilitate design of effective multiple trait selection schemes.

## Methods

### Data simulation

Simulated data, which resembled the situation encountered in the population of the Hanoverian Warmblood horse, were used for this study. Characteristics of this population include a high percentage of artificial insemination and coexistence of many small breeding farms and few large studs. The number of horses used for breeding is rather low when compared to the total number of horses, but data collection for genetic analyses is not limited to broodmares and sires. We simulated fixed effects, residual and additive genetic variances for one continuous trait (T1) and liabilities of four categorical traits (T2 to T5), and QTL effects for the liability of one of the categorical traits (T2). Heritabilities were set to 0.50 (T1), 0.25 (T3, T5) and 0.10 (T2, T4). Simulated additive genetic correlations were positive between T1 and T2 and between T1 and T3 (r_g _= 0.20), and negative between T1 and T4 and between T4 and T5 (r_g _= -0.20). For T2 two QTL and two flanking markers per QTL with five randomly distributed and equally prevalent alleles each were simulated. Linkage between markers and corresponding QTL was assumed, with one of the marker alleles being associated with the unfavorable QTL allele, therewith indicating increased probability of affection with regard to T2. We assumed that the closely linked markers and the unfavorable allele of each of the markers had been identified in previous association studies, allowing the distinction between individuals homozygous positive, heterozygous or homozygous negative for the unfavorable allele of the respective marker. Only additive effects and no dominance effects were simulated, and total QTL variance was set equal to the polygenic variance. In order to study the effects of different quality of QTL information on the estimation of genetic parameters, three scenarios with respect to recombination rate between markers and QTL (r) and polymorphism information content (PIC) of markers were simulated: r = 0.00 and PIC = 0.9 for all markers (r0p9); r = 0.01 and PIC = 0.9 for all markers (r1p9); r = 0.00 and PIC = 0.7 for all markers (r0p7). In this context, a recombination rate of 0.01 between a marker and the QTL will mean that given the unfavorable marker allele, the QTL allele will be unfavorable with probability p = 0.99. A recombination rate of 0.00 between a marker and the QTL will mean that given the unfavorable marker allele, the QTL allele will be unfavorable with probability p = 1.00. This case is identical with the situation in which the causal mutation is definitely known. For the categorical traits, simulation of liabilities on the linear scale was followed by dichotomization in order to obtain trait prevalences of 25% (T_2_, T_5_) or 10% (T_3_, T_4_). Details on the simulation procedure are summarized in Table 5.

**Table 5:** Characteristics of the simulated animal populations.

Population parameter	Simulated value
Total number of animals	280000
Number of animals per generation	40000
Sex ratio (males : females)	1 : 1
Number of contemporary groups per generation	5 (with 2 contemporary groups each represented in 2 subsequent generations)
Number of breeding animals per generation	9400 (9000 dams/400 sires)
Number of offspring per dam or sire	dams: 5 sires: 500 (n = 40) or 150 (n = 160) or 5 (n = 200)
Distribution of additive genetic effects	a_1 _~ N(0, 4.8) and a_2–5 _~ N(0, 1.0) with r_g12 _= r_g13 _= 0.20 and r_g14 _= r_g45 _= -0.20 and a_offspring _= 0.5 (a_sire _+ a_dam_) + m
Distribution of residual effects	e ~ N(0, x) with x so that h^2^_1 _= 0.50, h^2^_2 _= h^2^_4 _= 0.10, and h^2^_3 _= h^2^_5 _= 0.25
Prevalences of binary traits after dichotomization	T_2_, T_5_: 0.25, and T_3_, T_4_: 0.10
Number of QTL (T2)	2
Number of genetic marker per QTL	2
Number of alleles per genetic marker	5
Recombination rate between markers and QTL	0.0 or 0.1
PIC of genetic markers	0.9 or 0.7

a_i_: random additive genetic effect for trait i (i = 1–5); r_gij_: additive genetic correlation between traits i and j (i = j = 1–5); a_offspring _(a_sire_, a_dam_): additive genetic effect of offspring (sire, dam); m: Mendelian sampling term with m ~ N(0, 0.5σ_ai_); e: random residual; h^2^_i_: heritability for trait i (i = 1–5; overall heritability for trait 2);

QTL: quantitative trait locus; PIC: polymorphism information content.

Each of the three simulated populations included 7 generations and 40000 animals per generation. Samples of 10000 animals were drawn at random from the fourth generation, and the pedigree of these animals was traced back over three generations. Within each of ten replicates which were generated this way, six different datasets were created for the genetic analyses. Dataset A1 included all 10000 animals with records for the continuous trait and the four binary traits, information on the fixed effects of sex and contemporary group, and pedigree information over three generations. Dataset B1 included 5000 animals, randomly chosen from the animals included in dataset A1, with respective information on traits, sex, contemporary group and pedigree. Dataset C1 included the same animals and the same information as dataset B1, and additional information on the marker genotype of the animals. Dataset B2 included the same animals as datasets B1 and C1 plus their parents with information on traits, sex, contemporary group and pedigree. Dataset C2 included the same animals and the same information as dataset B2, and additional information on the marker genotype of the animals. Dataset A2 included the same animals as datasets B2 and C2 plus the additional 5000 animals which were included in dataset A1 with information on traits, sex, contemporary group and pedigree. The average size of paternal halfsib groups ranged between 16.30 and 29.28, and the average size of maternal halfsib groups ranged between 1.23 and 1.55 among the animals with trait records in the six datasets. Distribution of trait and pedigree information in the different datasets used for the genetic analyses is summarized in Table 6.

**Table 6:** Mean, minimum and maximum of trait and pedigree information in the six datasets generated within each of ten replicates.

	Dataset			
	
Population parameter	A1	A2	B1, C1	B2, C2
Animals_phen_	10000	14289 (14236–14317)	5000	9289 (9236–9317)
Prev_T2_	25.32 (24.78–25.71)	25.62 (25.38–25.87)	25.18 (24.36–25.72)	25.82 (25.24–26.37)
Prev_T3_	9.92 (9.49–10.44)	9.52 (9.18–10.06)	9.87 (9.10–10.44)	9.31 (8.80–9.80)
Prev_T4_	9.53 (9.09–10.11)	9.81 (9.43–10.06)	9.50 (8.96–10.32)	9.92 (9.55–10.32)
Prev_T5_	26.00 (25.29–26.84)	25.11 (24.37–25.75)	25.91 (24.84–27.08)	24.52 (23.58–25.29)
Animals_gen_	0	0	B1: 0 C1: 5000	B2: 0 C2: 9289 (9236–9317)
Animals_total_	30642 (30533–30766)	37185 (37028–37292)	19913 (19791–20050)	26457 (26253–26664)
Sires	342 (333–352)	625 (614–634)	287 (273–303)	570 (557–585)
HSG_pat_	29.28 (1–352)	22.88 (1–135)	17.43 (1–81)	16.30 (1–81)
Dams	6448 (6403–6489)	10001 (9936–10052)	4002 (3946–4033)	7556 (7483–7619)
HSG_mat_	1.55 (1–5)	1.43 (1–5)	1.25 (1–5)	1.23 (1–5)

Animals_phen_: number of animals with phenotypic trait records; Prev_T2 _(Prev_T3_, Prev_T4_, Prev_T5_): prevalence of binary trait T2 (T3, T4, T5); Animals_gen_: number of animals with genetic marker information; Animals_total_: total number of animals in the relationship matrix; Sires (Dams): number of sires (dams) of animals with phenotypic trait records; HSG_pat _(HSG_mat_): size of paternal halfsib groups among the animals with phenotypic trait records

### Statistical analyses

Genetic parameters were estimated using Gibbs sampling with the threshold version of the Multiple Trait Gibbs Sampler for Animal Models (MTGSAM) [35], a software which supports multivariate genetic analyses of any combination of continuous and categorical traits. Random and residual effects are assumed to be normally distributed, and flat priors are used for the fixed effects. The user can specify starting values and priors for additive genetic and residual (co)variance matrices. For our analyses, a starting value of one was chosen for all additive genetic variances, a starting value of zero was chosen for all additive genetic covariances, and the residual covariances between all traits were fixed to zero. In uni- and multivariate binary threshold models identifiability of the model is ensured by fixing the values for the thresholds and the residual variances to values of zero and one, respectively [36]. Methods for effective sampling of the residual covariance matrix subject to the restriction of diagonal elements fixed at one are still under development. However, fixation of the residual covariances for this study was justified by the results of previous real data analyses [14] and fit our simulated data. Because residual covariances were negligible in the real data and were accordingly set to zero in the data simulation, possible gain of further information seemed to be disproportionate to the additional costs of sampling of the residual covariances. Residual covariances were therefore fixed to zero in this study. For the genetic covariance matrix a proper prior using an inverse Wishart distribution with minimum shape parameter (i.e. ν_IW _= 7) was adopted in order to ensure posterior propriety. The fixed effects of sex and contemporary group were considered in all analyses. The fixed effect of marker genotype was considered in the analyses of datasets C1 and C2 only, distinguishing between individuals homozygous negative for the unfavorable alleles of all genetic markers, individuals heterozygous for the unfavorable allele of at least one of the genetic markers, and individuals homozygous for the unfavorable allele of at least one of the four genetic markers.

*y*_*ijlm *_= *μ *+ *SEX*_*i *_+ *CONT*_*j *_+ *a*_*l *_+ *e*_*ijlm*_(datasets A1, A2, B1 and B2), and

*y*_*ijklm *_= *μ *+ *SEX*_*i *_+ *CONT*_*j *_+ *QTL*_*k *_+ *a*_*l *_+ *e*_*ijklm*_(datasets C1 and C2),

with *y*_*ijlm*_(*y*_*ijklm*_) = observation on trait T1 (continuous) or on trait T2, T3, T4 or T5 (binary) for the l^th ^animal,

*μ *= model constant,

*SEX*_*i *_= fixed effect of the sex of the animal (i = 1–2),

*CONT*_*j *_= fixed effect of the contemporary group (j = 1–5 in analyses of datasets A1, B1 and C1; j = 1–8 in analyses of datasets A2, B2 and C2),

*QTL*_*k *_= fixed effect of the QTL marker genotype (n = 1–3),

*a*_*l *_= random additive genetic effect of the l^th ^animal (l = 1–30533 to 30766 for dataset A1, l = 1–37028 to 37292 for dataset A2, l = 1–19791 to 20050 for datasets B1 and C1, and l = 1–26253 to 26664 for datasets B2 and C2), and

*e*_*ijlm*_(*e*_*ijklm*_) = random residual.

The total length of the Gibbs chain was set to 205000 in all analyses, and all samples after 5000 rounds of burn-in were saved. Convergence of the Gibbs chain and the need for additional rounds of burn-in to be discarded was checked by visual inspection of sample plots. Effective sample size (ESS) and Monte Carlo error (MCE) was calculated for all (co)variance estimates by the times series method implemented in the post-Gibbs analysis program POSTGIBBSF90 [37] with a thinning rate of ten. Un-thinned chains were used to calculate posterior means of additive genetic (co)variance, heritability and additive genetic correlation estimates. Posterior means rather than modes were chosen as point estimates, because in preliminary analyses the means were in most cases closer to the true, i.e. simulated, values than the modes, a finding which is in agreement with previous studies [8,38]. Bias of heritability and additive genetic correlation estimates was calculated as the mean relative deviation of the estimated values (par_est_) from the true, i.e. simulated, values (par_true_).

*bias *= (*par*_*est *_- *par*_*true*_)/*par*_*true*_

The influence of data structure and quality of genetic marker information on ESS and bias was tested via analysis of variance using the procedure GLM of Statistical Analysis Systems, (SAS), version 9.1.3 (SAS Institute, Cary, NC, USA, 2005). Effective sample size or bias were considered as dependent variable, and dataset (A1, A2, B1, B2, C1, C2) and quality of genetic marker information (r0p9, r1p9, r0p7) were considered as fixed effect.

## Authors' contributions

KFS designed and carried out the simulation study, and drafted the manuscript. OD conceived of the study, participated in its design, and revised the manuscript. IH participated in conception and design of the study, coordinated it, and revised the manuscript. All authors read and approved the final manuscript.

## References

[B1] Abdel-Azim GA, Berger PJ (1999). Properties of threshold model predictions. Journal of Anim Sci.

[B2] Mäntysaari EA, Quaas RL, Gröhn YT (1991). Simulation study on covariance component estimation for two binary traits in an underlying continuous scale. J Dairy Sci.

[B3] Van Vleck LD (1972). Estimation of heritability of threshold characters. J Dairy Sci.

[B4] Van Vleck LD, Gregory KE (1992). Multiple-trait restricted maximum likelihood for simulated measures of ovulation rate with underlying multivariate normal distributions. J Anim Sci.

[B5] Hoeschele I, Tier B (1995). Estimation of variance components of threshold characters by marginal posterior modes and means via Gibbs Sampling. Genet Sel Evol.

[B6] Matos CAP, Thomas DL, Gianola D, Tempelman RJ, Young LD (1997). Genetic analysis of discrete reproductive traits in sheep using linear and nonlinear models. I. Estimation of genetic parameters. J Anim Sci.

[B7] Meijering A, Gianola D (1985). Linear versus nonlinear methods of sire evaluation for categorical traits: a simulation study. Gen Sel Evol.

[B8] Luo MF, Boettcher PJ, Schaeffer LR, Dekkers JCM (2001). Bayesian inference for categorical traits with an application to variance component estimation. J Dairy Sci.

[B9] Misztal I, Gianola D, Foulley JL (1989). Computing aspects of a nonlinear method of sire evaluation for categorical data. J Dairy Sci.

[B10] Moreno C, Sorensen D, García-Cortés LA, Varona L, Altarriba J (1997). On biased inferences about variance components in the binary threshold model. Gen Sel Evol.

[B11] Jensen J Bayesian analysis of bivariate mixed models with one continuous and one binary trait using the Gibbs Sampler. Proceedings of the 5th World Congress on Genetics Applied to Livestock Production: 7–12 August 1994.

[B12] Simianer H, Schaeffer LR (1989). Estimation of covariance components between one continuous and one binary trait. Gen Sel Evol.

[B13] Wang CS, Quaas RL, Pollak EJ (1997). Bayesian analysis of calving ease scores and birth weights. Gen Sel Evol.

[B14] Stock KF, Distl O (2006). Genetic correlations between osseous fragments in fetlock and hock joints, deforming arthropathy in hock joints and pathologic changes in the navicular bones of Warmblood riding horses. Livest Sci.

[B15] Stock KF, Distl O (2006). Genetic analyses of radiographic appearance of the navicular bones in the Warmblood horse. Am J Vet Res.

[B16] Willms F, Röhe R, Kalm E (1999). Genetische Analyse von Merkmalskomplexen in der Reitpferdezucht unter Berücksichtigung von Gliedmaßenveränderungen. 1. Mitteilung: Züchterische Bedeutung von Gliedmaßenveränderungen. Züchtungskunde.

[B17] Winter D, Bruns E, Glodek P, Hertsch B (1996). Genetische Disposition von Gliedmaßenerkrankungen bei Reitpferden. Züchtungskunde.

[B18] Stock KF, Distl O (2005). Prediction of breeding values for osseous fragments in fetlock and hock joints, deforming arthropathy in hock joints and pathologic changes in navicular bones of Hanoverian Warmblood horses. Livest Prod Sci.

[B19] Stock KF, Distl O (2005). Expected response to selection when accounting for orthopedic health traits in a population of Warmblood riding horses. Am J Vet Res.

[B20] Dempster ER, Lerner IM (1950). Heritability of threshold characters. Genetics.

[B21] Vinson WE, White JM, Kliewer RH (1976). Overall classification as a selection criterion for improving categorically scored components of type in Holsteins. J Dairy Sci.

[B22] Stock KF, Hamann H, Distl O (2005). Estimation of genetic parameters for the prevalence of osseous fragments in limb joints of Hanoverian Warmblood horses. J Anim Breed Genet.

[B23] Dekkers JCM (2004). Commercial application of marker- and gene-assisted selection in livestock: strategies and lessons. J Anim Sci (E Suppl).

[B24] Böneker C, Löhring K, Wittwer C, Distl O Genome-wide search for markers associated with osteochondrosis in Hanoverian Warmblood horses.

[B25] Dierks C, Distl O Identification of a quantitative gene locus on equine chromosome 4 responsible for osteochondrosis in hock joints of Hanoverian Warmblood horses.

[B26] Lande R, Thompson R (1990). Efficiency of marker-assisted selection in the improvement of quantitative traits. Genetics.

[B27] Brka M, Reinsch N, Kalm E (2002). Frequency and heritability of supernumerary teats in German Simmental and German Brown Swiss cows. J Dairy Sci.

[B28] Hansen M, Lund MS, Pedersen J, Christensen LG (2004). Genetic parameters for stillbirth in Danish Holstein cows using a Bayesian threshold model. J Dairy Sci.

[B29] Lund MS, Jensen J, Petersen PH (1999). Estimation of genetic and phenotypic parameters for clinical mastitis, somatic cell production deviance, and protein yield in dairy cattle using Gibbs sampling. J Dairy Sci.

[B30] Piles M, Rafel O, Ramon J, Varona L (2005). Genetic parameters of fertility in two lines of rabbit of different reproductive potential. J Anim Sci.

[B31] Janssens S, Vandepitte W, Bodin L (2004). Genetic parameters for litter size in sheep: natural versus hormone-induced oestrus. Gen Sel Evol.

[B32] Hoeschele I, Gianola D, Foulley JL (1987). Estimation of variance components with quasi-continuous data using Bayesian methods. J Anim Breed Genet.

[B33] Gates P, Johansson K, Danell B (1999). "Quasi-REML" correlation estimates between production and health traits in the presence of selection and confounding: a simulation study. J Anim Sci.

[B34] Gianola D (1982). Theory and analysis of threshold characters. J Anim Sci.

[B35] Van Tassell CP, Van Vleck LD (1996). Multiple-trait Gibbs sampler for animal models: flexible programs for Bayesian and likelihood-based (co)variance component inference. J Anim Sci.

[B36] Harville DA, Mee RW (1984). A mixed-model procedure for analyzing ordered categorical. Biometrics.

[B37] Tsurata S POSTGIBBSF90. http://nce.ads.uga.edu/~ignacy/newprograms.html.

[B38] Van Tassell CP, Casella G, Pollak EJ (1995). Effects of selection on estimates of variance components using Gibbs sampling and Restricted Maximum Likelihood. J Dairy Sci.

